# Veterinary Research reviewer acknowledgement 2013

**DOI:** 10.1186/1297-9716-45-4

**Published:** 2014-01-20

**Authors:** Michel Brémont, Élodie Coulamy

**Affiliations:** 1 Veterinary Research, Unité de Virologie et Immunologie Moléculaires, INRA, Domaine de Vilvert, 78352 Jouy-en-Josas, Cedex, France

## Abstract

**Contributing reviewers:**

The *Veterinary Research* editorial team would sincerely like to thank all of our reviewers who contributed to peer review for the journal in 2013.

## 

Darrell Abernethy

South Africa

Joana Abrantes

Portugal

Mathias Ackermann

Switzerland

Pier Luigi Acutis

Italy

Diane Addie

France

Tahar Ait-Ali

United Kingdom

Sonia Almería

Spain

Nick Andronicos

Australia

Denis Archambault

Canada

Fabienne Archer

France

Ellen Ariel

Australia

Brian Austin

United Kingdom

Jantien Backer

Netherlands

Susan Baigent

United Kingdom

Ernest Bailey

United States of America

Cynthia Baldwin

United States of America

Keith Ballingall

United Kingdom

Thierry Baron

France

Paul Bartley

United Kingdom

Andrew Bean

Australia

Shawn Bearson

United States of America

Ingeborg Becker

Mexico

Martin Beer

Germany

Julio Benavides

Spain

Marie-Odile Benoit-Biancamano

Canada

Mikael Berg

Sweden

Jean-François Bernardet

France

Giuseppe Bertoni

Switzerland

Camilla Björkman

Sweden

Barbara Blacklaws

United Kingdom

Damer Blake

United Kingdom

David Blehert

United States of America

Sanne Charles Bodjo

Ethiopia

Karl Boehme

United States of America

Josef Böhm

Austria

Belen Borrego

Spain

Pierre Boudinot

France

Thierry Boulinier

France

Richard Bowen

United States of America

Filip Boyen

Belgium

Paulo Brandão

Brazil

Samantha Brandler

France

Sabine Brandt

Austria

Romulus Breban

France

Jennifer Brisbin

Canada

Earl Brown

Canada

Wendy Brown

United States of America

Craig Brunetti

Canada

Bryce Buddle

New Zealand

Thomas Burkey

United States of America

Christian Burvenich

Belgium

Patrick Butaye

Belgium

David Buxton

United Kingdom

Lorenzo Capucci

Italy

Alexandre Caron

Zimbabwe

Consuelo Carrillo

United States of America

Jordi Casal

Spain

José María Castro

Spain

Isabella Cattadori

United States of America

Giovanni Cattoli

Italy

Shaun Cawthraw

United Kingdom

Brigitte Cay

Belgium

Chanhee Chae

Korea, South

Bryan Charleston

United Kingdom

Bernard Charley

France

Yahia Chebloune

France

Véronique Chevalier

France

Bruno Chomel

United States of America

Rémi Choquet

France

Axel Cloeckaert

France

Ellen Collisson

United States of America

Richard Cook

United States of America

Chris Cousens

United Kingdom

Paul Coussens

United States of America

Eric Cox

Belgium

Siska Croubels

Belgium

Charles Czuprynski

United States of America

Sirlei Daffre

Brazil

Ginette Dambrine

France

Andrew Davison

United Kingdom

Jeroen De Buck

Canada

Henri De Greve

Belgium

Aalzen de Haan

Netherlands

Sarne De Vliegher

Belgium

Annemie Decostere

Belgium

Cornelia Deeg

Germany

Aldo Dekker

Netherlands

Caroline Denesvre

France

Pratima Deshpande

United States of America

Adama Diallo

Austria

Isabelle Dimier-Poisson

France

Linda Dixon

United Kingdom

Steven Djordjevic

Australia

Javier Domínguez Juncal

Spain

Charles Martin Dozois

Canada

Elizabeth Driskell

United States of America

Richard Ducatelle

Belgium

Mariette Ducatez

France

Luc Duchateau

Belgium

Ildiko Rita Dunay

Germany

Herman Egberink

Netherlands

William Ellis

United Kingdom

Gary Entrican

United Kingdom

Kevin Esch

United States of America

Pedro Esteves

Portugal

Øystein Evensen

Norway

Olivia Faulkner

United States of America

Richard Ferrero

Australia

Nahuel Fittipaldi

United States of America

Neil Foster

United Kingdom

Gilles Foucras

France

Lorenzo Fraile

Spain

Arthur Frampton

United States of America

Klaas Frankena

Netherlands

Joachim Frey

Switzerland

Winifred Frick

United States of America

Walter Fuchs

Germany

João Luis Garcia

Brazil

Oliver Garden

United Kingdom

Robin Gasser

Australia

Peter Geldhof

Belgium

Volker Gerdts

Canada

Wilhelm Gerner

Austria

Souvik Ghosh

Japan

Robert Gilbert

United States of America

Sarah Gilbert

United Kingdom

John Gilleard

Canada

Liz Glass

United Kingdom

William Golde

United States of America

Wilfred Goldmann

United Kingdom

Christian Gortazar

Spain

Marcelo Gottschalk

Canada

Kaare Græsbøll

Denmark

Simon Graham

United Kingdom

Beatrice Grasland

France

Andrew Greer

New Zealand

Philip Griebel

Canada

Marvin J. Grubman

United States of America

Guiquan Guan

China

Luca Guardabassi

Denmark

Simon Gubbins

United Kingdom

Laurence Guzylack-Piriou

France

Carlton Gyles

Canada

Bernd Haas

Germany

Sonja Härtle

Germany

Freddy Haesebrouck

Belgium

Chris Hall

United States of America

David Hampson

Australia

Balazs Harrach

Hungary

Christopher Heaney

United States of America

Andrew Hemphill

Switzerland

Guy Hendrickx

Belgium

Jesús Hernández

Mexico

Marc Heyndrickx

Belgium

Ludwig Eduard Hölzle

Germany

Johan Höglund

Sweden

Ole Højberg

Denmark

Cornelis Hokke

Netherlands

Geoffrey Holm

United States of America

Jayne Hope

United Kingdom

John Hopkins

United Kingdom

Emily Hotchkiss

United Kingdom

Kris Huygen

Belgium

Elisabeth Innes

United Kingdom

Ilgiz Irnazarow

Poland

Mario Jacques

Canada

Véronique Jestin

France

Sven Martin Jørgensen

Norway

Tae Sung Jung

Korea, South

Gregers Jungersen

Denmark

Tobias Kaeser

Austria

Pete Kaiser

United Kingdom

Isis Kanevsky-Mullarky

United States of America

Bernd Kaspers

Germany

Barbara Katzenback

Canada

Frank Katzer

United Kingdom

David Kerr

United States of America

Anthony Keyburn

Australia

Seong Kim

United States of America

Su-Mi Kim

Korea, South

Takashi Kimura

Japan

Peter Kirkland

Australia

Marcel Klaassen

Australia

Siv Klevar

Norway

Bernard Klonjkowski

France

Michael Kogut

United States of America

Nicholas Komar

United States of America

Petr Kopacek

Czech Republic

Kelly Lager

United States of America

Päivi Lahti

Falkland Islands

Jan Langeveld

Netherlands

Scott LaPatra

United States of America

Lars Larsen

Denmark

Ronan Le Goffic

France

Cyril Le Nouen

United States of America

Jacques Le Pendu

France

Marie-Frederique Le Potier

France

Sylvie Lecollinet

France

François Lefèvre

France

Caroline Leroux

France

Robert Li

United States of America

Geneviève Libeau

France

Niels Lorenzen

Denmark

Bertrand Losson

Belgium

Joan Lunney

United States of America

Blanca Lupiani

United States of America

N James Maclachlan

United States of America

Dominiek Maes

Belgium

Jeffrey Mariner

Kenya

Raquel Marques

Portugal

An Martel

Belgium

Vito Martella

Italy

Paolo Martelli

Italy

Silvia Martinez-Subiela

Spain

Carlos Martins

Portugal

Pascale Massin

France

Enric Mateu

Spain

Simonetta Mattiucci

Italy

James McNair

United Kingdom

Tom McNeilly

United Kingdom

Francois Meurens

Canada

Evelyne Meyer

Belgium

Tiago Mineo

Brazil

Ronald Montelaro

United States of America

Maria Montoya

Spain

Arshnee Moodley

Denmark

Robert Moore

Australia

Simon More

Ireland

Ana Moreno

Italy

Kate Mounsey

Australia

Michael Murtaugh

United States of America

Nadia Naffakh

France

Lubna Nasir

United Kingdom

Hans Nauwynck

Belgium

Paulina Niedzwiedzka-Rystwej

Poland

Alasdair Nisbet

United Kingdom

Marinda Oosthuizen

South Africa

Tanja Opriessnig

United States of America

Luis Miguel Ortega-Mora

Spain

Fernando A. Osorio

United States of America

Antonio Osuna

Spain

Isabelle Oswald

France

Mary Pantin-Jackwood

United States of America

Robert Parkhouse

Portugal

Frank Pasmans

Belgium

B Pattnaik

India

Niels Pedersen

United States of America

Luc Peelman

Belgium

Ben Peeters

Netherlands

Sebastien Plumet

France

John Prescott

Canada

Lance Price

United States of America

Cinta Prieto

Spain

Qiwei Qin

China

Melvyn Quan

South Africa

Pascal Rainard

France

Gustavo Real

Spain

William Reisen

United States of America

Xiaofeng Ren

China

Gourapura Renukaradhya

United States of America

Jack Rhyan

United States of America

Jürgen Richt

United States of America

Gabriele Rieder

Austria

Bertus Rima

United Kingdom

Espen Rimstad

Norway

Jacques Robert

United States of America

Julian Rood

Australia

Mirko Rossi

Finland

Peter J.M. Rottier

Netherlands

Nicolas Ruggli

Switzerland

Saskia Rutjes

Netherlands

Ivan Rychlik

Czech Republic

Armin Saalmüller

Austria

Claude Saegerman

Belgium

Siba Samal

United States of America

Jose Sánchez-Vizcaíno

Spain

Gereon Schares

Germany

Horst Schirrmeier

Germany

Leonhard Schnittger

Argentina

Isabelle Schwartz

France

Luigi Sedda

United Kingdom

Sang Heui Seo

Korea, South

Melkote Shaila

India

Shayan Sharif

Canada

Susan Shriner

United States of America

Gaëlle Simon

France

Alf Skovgaard

Denmark

Chantel Sloan

United States of America

Annemieke Smet

Belgium

Natalia Smirnova

United States of America

Francisco Sobrino

Spain

Chang-Seon Song

Korea, South

Erin Sorrell

United States of America

Allyn Spear

United States of America

Judith Stabel

United States of America

Michael Stack

United Kingdom

Katharina Staerk

Switzerland

Glyn Stanway

United Kingdom

Michael Stear

United Kingdom

Jürgen Stech

Germany

Arjan Stegeman

Netherlands

Dieter Steinhagen

Germany

Mark Stevens

United Kingdom

Karen Stevenson

United Kingdom

Norbert Stockhofe-Zurwieden

Netherlands

Anne K Storset

Norway

Tanja Strive

Australia

Rebecca Strong

United Kingdom

Artur Summerfield

Switzerland

Agus Sunarto

Australia

Ian Sutherland

New Zealand

Jun Suzuki

Japan

Raymond Sweeney

United States of America

Fumihiro Taguchi

Japan

Geraldine Taylor

United Kingdom

Eileen Thacker

United States of America

Keith Thoday

United Kingdom

Leen Timbermont

Belgium

Richard Titball

United Kingdom

Michael Andreas Tranulis

Norway

Wenbin Tuo

United States of America

Tobias Tuthill

United Kingdom

Stephen Valas

France

Gerlinde Van de Walle

United States of America

Tom Van de Wiele

Belgium

Thierry van den Berg

Belgium

Wim van der Poel

Netherlands

Mirjam van der Sluijs

Netherlands

Filip Van Immerseel

Belgium

Alain Vanderplasschen

Belgium

Mario Vaneechoutte

Belgium

Daisy Vanrompay

Belgium

Philippe Velge

France

Koen Verstappen

Netherlands

Pierre-Olivier Vidalain

France

Victoriya Volkova

United States of America

Vladimir Vrba

Czech Republic

Paul Walz

United States of America

Michael Ward

Australia

Jonathan Wastling

United Kingdom

Scott Weese

Canada

Rudi Weiblen

Brazil

Adrian Whitehouse

United Kingdom

Gary Whittaker

United States of America

Diana Williams

United Kingdom

Kyoung-Jin Yoon

United States of America

Guofan Zhang

China

Xing-Quan Zhu

China

Jun Zou

United Kingdom

Federico Zuckermann

United States of America

